# Use of the Screening for Occult Renal Disease (SCORED) Questionnaire to Screen Diabetic Nephropathy in Asymptomatic Patients: A Cross-Sectional Study

**DOI:** 10.7759/cureus.66327

**Published:** 2024-08-06

**Authors:** Flávio Augusto P Aleixo, Luíza Israel S Assunção, Matheus Lucca Ângelo C Rodrigues, Laura Lisboa O Vieira, Vitor Hugo M Vilela, Cristiane R Correa

**Affiliations:** 1 Surgery, Faculdade de Ciências Médicas de Minas Gerais, Belo Horizonte, BRA; 2 Internal Medicine, Faculdade de Ciências Médicas de Minas Gerais, Belo Horizonte, BRA; 3 Internal Medicine, Universidade Federal de Minas Gerais, Belo Horizonte, BRA; 4 Biochemistry, Faculdade de Ciências Médicas de Minas Gerais, Belo Horizonte, BRA

**Keywords:** chronic kidney disease, nephropathy, kidney, diabetes, scored

## Abstract

Introduction: The 2007 Screening for Occult Renal Disease (SCORED) questionnaire accesses risk factors for chronic kidney disease (CKD) and makes it possible to screen high-risk patients, being adapted and validated for the Brazilian culture in 2012. The present study evaluated the questionnaire's ability to predict the occurrence of CKD in asymptomatic patients, as well as identify a high risk for developing the disease.

Methods: This was an analytical observational study with a cross-sectional design carried out in two stages: answering the SCORED and performing fasting blood glucose and creatinine tests. The participants were patients at the Hospital das Clínicas of the Federal University of Minas Gerais (HC-UFMG) with scheduled creatinine and fasting blood glucose tests, respecting the inclusion and exclusion criteria defined for the study. SCORED was applied with questions covering gender, age, proteinuria, and diabetes, being classified as high or low risk for CKD. The data collected were height, weight, age, sex, diagnosis of diabetes mellitus, and fasting blood glucose.

Results: The sample space was 212 individuals, the majority of whom were female (N=130, 61.3%), with a median of 58 years of age. The prevalence of CKD was 15.6% (N=33) with a sensitivity of 90.9%, a specificity of 30.2%, a positive predictive value of 19.4%, a negative predictive value of 94.7%, and a diagnostic accuracy of 39.7%.

Conclusion: We concluded that the SCORED questionnaire can be a useful tool for screening CKD in asymptomatic patients and also that there is a relationship was detected between glycemic changes and an increased risk for CKD.

## Introduction

Chronic kidney disease (CKD) is a clinical syndrome characterized by structural abnormalities and progressive loss of kidney function, resulting in consequences for the patient's health [1]. The Kidney Disease: Improving Global Outcomes (KDIGO) [2] organization defines CKD as a persistent reduction in glomerular filtration rate (GFR) below 60 ml/min/1.73 m², the presence of at least one marker of kidney parenchymal injury, or the association of both, for at least three months [3].

Diabetes mellitus (DM) is the leading cause of chronic and end-stage renal disease in developed countries [4]. Approximately, 20-40% of DM patients will develop diabetic nephropathy (DN) [5], a microvasculopathy in the renal arteries that initially manifests as albuminuria and progressively evolves with declining renal function, representing the natural history of CKD in these individuals. The presentation can occur 5-15 years after the diagnosis of type 1 diabetes and can already be present at the diagnosis of type 2 diabetes [5].

In 2007, the Screening for Occult Renal Disease (SCORED) questionnaire was published, which evaluates CKD risk factors and enables the screening of high-risk patients. Asymptomatic individuals with abnormal questionnaire results would have a 20% chance of being CKD patients [6]. In 2012, SCORED was adapted and validated for the Brazilian culture [7].

The present study aims to primarily analyze the ability of SCORED to predict the occurrence of CKD in asymptomatic patients and evaluate the identification of individuals at high risk for disease development, comparing the results with the questionnaire validation study in Brazil. Secondary objectives include stratifying CKD risk based on isolated risk factors (altered fasting glucose) and assessing the possibility of using the test for CKD screening in asymptomatic patients.

## Materials and methods

This is an analytical observational study with a cross-sectional design. It was submitted to and approved by the Research Ethics Committee of the Faculdade de Ciências Médicas de Minas Gerais (CEPCM-MG) (approval number: 1.326.113). It followed the Strengthening the Reporting of Observational Studies in Epidemiology (STROBE) guidelines for cross-sectional studies [6], and participants signed the informed consent form (ICF).

The sample was non-randomly selected for convenience, and volunteers were patients from the Hospital das Clínicas of the Federal University of Minas Gerais (HC-UFMG), scheduled for fasting blood glucose and creatinine tests. Inclusion criteria were as follows: being over 18 years old or having parental or legal guardian consent, attending the laboratory for the tests (evaluated using the international standardized isotope dilution mass spectrometry (IDMS)), and signing the ICF.

The SCORED [6] questionnaire was applied to identify individuals with a high likelihood of having occult renal disease. The questionnaire consists of 12 questions covering topics such as sex, age, urinary protein loss, and diabetes. According to the test validation, if the patient scores 4 or more points, they have a 20% chance of having CKD. If they score 0-3, they probably do not have kidney disease now, but screening should be done at least once a year.

Patients who scored equal to or higher than 4 on the SCORED questionnaire were considered at high risk for CKD, and subsequently, the prevalence of CKD risk factors and renal function were compared using creatinine clearance (CrCl).

All patients or guardians received and signed the ICF, which explained the study's objectives, conditions, and volunteer rights. Once they agreed to participate in the research, three steps were followed: data collection; completion of the proposed questionnaire, SCORED; and performance of the tests. The collected data included age, sex, previous diagnosis of DM, height, and weight for body mass index (BMI) calculation.

The CKD-EPI formula was used to calculate CrCl, defining CKD patients as those with a filtration rate lower than 60 mL/min/1.73 m².

The patients were divided in three different ways. The first considered scores higher than 4 or lower than 3 on the SCORED. The second evaluated only fasting glucose levels according to the following ranges: <100 mg/dL, between 100 and 126 mg/dL, and >126 mg/dL. And the third analyzed protein and creatinine tests equal to or higher than 1.3 mg/dL or lower than 1.3 mg/dL. Groups were compared to establish a relationship between the studied variables.

Data analysis

Categorical variables were expressed as percentages, while continuous variables were expressed as medians and interquartile ranges, after checking the distribution mode in the population using the Kolmogorov-Smirnov test. For statistical comparison of categorical variables, the chi-squared test or Fisher's exact test was used, as appropriate. For continuous variables, the Mann-Whitney U test was used, using IBM SPSS Statistics for Windows, Version 23.0 (Released 2015; IBM Corp., Armonk, New York, United States).

## Results

The study included 212 individuals, whose risk factors for CKD, SCORED questionnaire scores, and serum creatinine and fasting glucose tests were evaluated (Table 1). The majority were female (N=130, 61.3%) with a median age of 58 years. The main comorbidities found were diabetes, in 36.8% (N=78) of the sample, and obesity (considering BMI ≥30), in 24.1% (N=51). Analysis of the tests showed serum creatinine equal to or higher than 1.3 mg/dL in 9.9% of individuals (N=21) and glucose ≥100 mg/dL in 44.3% (N=94). The SCORED questionnaire showed a high risk of developing CKD in 73.1% of individuals, i.e., 155 individuals (Table 1).

**Table 1:** Distribution of categorical and continuous variables (N=212) ¹Score on the SCORED ²Self-declared data SCORED: Screening for Occult Renal Disease; BMI: body mass index

Categorical and continuous variables	N	%
Female	130	61.3
Age ≥60 years	89	42
SCORED¹ ≥4	155	73.1
BMI ≥30	51	24.1
Fasting blood glucose ≥100 mg/dL	94	44.3
Fasting blood glucose ≥126 mg/dL	44	20.8
Fasting blood glucose between 100 and 126 mg/dL	50	23.6
Family history of kidney disease²	74	34.9
Past history of kidney disease²	52	24.5
Diabetes mellitus²	78	36.8
Proteinuria²	21	9.9
Creatinine ≥1.3	21	9.9

Table 2 demonstrates the proportion of patients with CKD (CrCl <60 mL/min/1.73 m²), from a total of 33 patients with a previously diagnosed CKD, and patients with a score equal to or higher than 4 on the SCORED. There is concordance between the variables.

**Table 2:** Comparison of categorical variables with the SCORED ≥4 and CKD groups (CrCl <60 mL/min/1.73 m²) ¹AFG: altered fasting glucose ²Self-declared data SCORED: score on the SCORED (Screening for Occult Renal Disease) questionnaire; CKD: chronic kidney disease; CrCl: creatinine clearance; CKD-EPI: glomerular filtration rate from the CKD epidemiology formula; BMI: body mass index

Variables	SCORED ≥4		CrCl <60 mL/min/1.73 m² - CKD-EPI	
	Yes		Yes	
	N	%	N	%
Blood glucose >100 mg/dL (AFG)¹	82	52.9	15	45.5
Current or previous smoking²	66	42.6	12	36.4
Diabetes mellitus²	123	79.4	26	78.8
Proteinuria²	14	9.0	5	15.2
Age ≥60 years	89	57.4	23	69.7
Age ≥70 years	34	21.9	11	33.3
Female	103	66.5	21	63.6
BMI ≥30	44	28.4	7	21.2

In Table 3, the relationship between the SCORED alteration and glycemic levels is observed. The median fasting glucose levels are significantly higher in the group at high risk of CKD.

**Table 3:** Fasting blood glucose values ​​according to the score on the SCORED SCORED: score on the SCORED (Screening for Occult Renal Disease) questionnaire

Variable	SCORED ≥4						
	No			Yes			
	Median	Percentile 25	Percentile 75	Median	Percentile 25	Percentile 75	p
Fasting blood glucose (mg/dL)	89	84	97	102	91	125	<0.001

To conduct a deeper analysis of the relationship between glycemic indices and the risk and development of renal disease, a separation was made between individuals with normal fasting glucose (NFG) with parameters <100 mg/dL and another group called altered fasting glucose, with values >100 mg/dL. The altered fasting glucose group had more patients with a score equal to or higher than 4 on the SCORED, with p<0.0001. Additionally, the glomerular filtration rate was relatively lower compared to NFG. When stratified from the diagnostic cutoff for DM, fasting glucose >126 mg/dL, a higher frequency of patients with altered SCORED was also corroborated, with p<0.017.

When comparing SCORED with the diagnosis by glomerular filtration rate (Table 4), there was correspondence in 90.9% of the 33 individuals with CKD, leading to the following conclusions. The SCORED has a sensitivity of 90.9% for CKD diagnosis, a specificity of 30.2%, a positive predictive value (PPV) of 19.4%, a negative predictive value (NPV) of 94.7%, and a diagnostic accuracy of 39.7%.

**Table 4:** High-risk patients for CKD (SCORED ≥4) versus CKD assessed by CKD-EPI SCORED: score on the SCORED (Screening for Occult Renal Disease) questionnaire; CrCl: creatinine clearance; CKD-EPI: glomerular filtration rate from the chronic kidney disease epidemiology formula; CKD: chronic kidney disease

Variable		CrCl <60 mL/min/1.73 m² - CKD-EPI	
		Yes	No
SCORED ≥4	Yes	30	125
	No	3	54

The final stratification of the study population showed that 155 out of 212 patients (73.1%) were at high risk of CKD according to the SCORED. Of these, 30 actually had CKD, totaling 33 patients diagnosed by CKD-EPI, i.e., a sensitivity of 90.9%.

**Figure 1:**
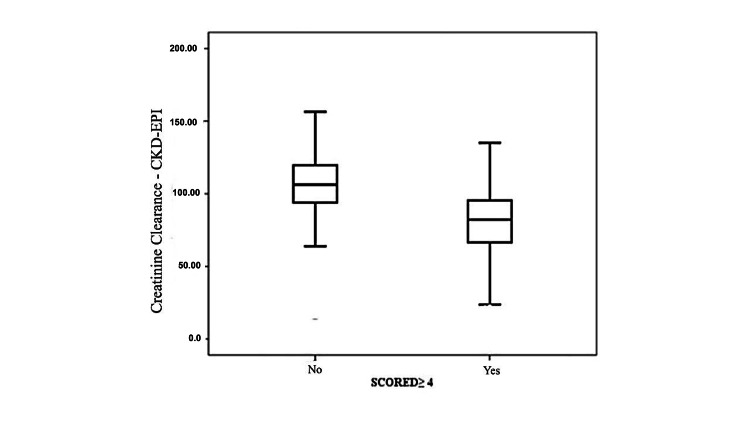
Relationship between creatinine clearance and the SCORED SCORED: Screening for Occult Renal Disease

Figure 1 shows the relationship between CrCl and the SCORED. There was a statistical difference between the groups, where the CrCl of patients at high risk for CKD was lower than that of patients with fewer risk factors for CKD.

## Discussion

Patients with CKD have a high rate of end-stage kidney disease, cardiovascular disease, and death, making CKD an appropriate target for prevention, early detection, and management by non-nephrologist clinicians and public health agencies [8,9]. The asymptomatic progression of CKD, particularly in its early stages, complicates diagnosis and often leads to loss of kidney function, development of complications, and increased morbidity and mortality [10].

In the present study, the prevalence of CKD (CrCl <60 mL/min/1.73 m²) was 15.6% (N=33), compared to the 10.6% prevalence reported by Magacho et al. [7] in the validation of the SCORED in Brazil. Both studies utilized volunteer patients from non-random convenience sampling. Our study analyzed users of outpatient clinics at the Hospital das Clínicas of the Federal University of Minas Gerais, while the Magacho et al. study evaluated the academic community of the Federal University of Juiz de Fora and their relatives.

The sample response to the SCORED and comparison with CrCl allowed for an analysis of the sensitivity, specificity, accuracy, PPV, and NPV of the SCORED. The base study validated the Brazilian SCORED [7] with a sensitivity of 80%, a specificity of 65%, a PPV of 14%, an NPV of 97%, and an accuracy of 66%. In contrast, our study found a sensitivity of 90.9%, a specificity of 30.2%, a PPV of 19.4%, an NPV of 94.7%, and a diagnostic accuracy of 39.7%.

The values obtained in our study align in some parameters with those found by Magacho et al. [7], particularly in terms of high sensitivity and NPV, but differ notably in specificity, PPV, and accuracy. These discrepancies may stem from differences in the study populations and settings. While both studies confirm the high sensitivity and NPV of the SCORED questionnaire, indicating its effectiveness in ruling out CKD, the variability in specificity highlights the need for cautious interpretation of positive results and suggests that further refinement of the questionnaire or additional screening criteria may be necessary to improve its diagnostic accuracy.

The analysis of variables separating patients according to their CrCl and CKD risk profile (score equal to or higher than 4) showed that the CrCl of patients at high risk for CKD is significantly lower than that of patients with a score lower than 4. This demonstrates that patients with more risk factors for CKD exhibit reduced renal function, even though most high-risk patients do not yet have clinically relevant CKD. The continuous presence of CKD risk factors accelerates disease progression, emphasizing the need for proactive monitoring and management of these patients to prevent deterioration [11]. Hence, patients identified as high risk by the SCORED questionnaire should be closely monitored, with appropriate treatment of their comorbidities to reduce the risk of progression to CKD.

Our comparison of glycemic groups with the questionnaire score revealed that the high-risk group for CKD (score equal to or higher than 4) had significantly higher fasting glucose levels than the lower-risk group (p<0.001). Hyperglycemia leads to the production of advanced glycation end products (AGE) and reactive oxygen species, which activate intracellular signaling pathways for proinflammatory and profibrotic gene expression, resulting in cellular injury [12,13]. This underscores the critical importance of glycemic control in patients with DM to delay the onset of CKD and other associated comorbidities.

Despite its low overall diagnostic accuracy, the high sensitivity (90.9%) and NPV (94.7%), combined with the low cost and ease of application, make the SCORED questionnaire a valuable tool for screening CKD at the population level, particularly in asymptomatic patients. This is especially relevant in health promotion campaigns or in resource-limited settings where access to medical care or specific diagnostic tests is challenging. Identifying high-risk individuals through the SCORED questionnaire enables early referral for medical evaluation and more specific diagnostic tests, potentially reducing the burden of CKD and associated cardiovascular diseases, which significantly impact healthcare costs [14,15].

The limitations of this study include the use of convenience sampling in a university healthcare setting, which may not be representative of the general population. Nevertheless, the study demonstrates the potential of the SCORED questionnaire as a screening tool, reinforcing its utility despite this limitation. Future research should focus on improving the specificity of the SCORED questionnaire and exploring additional factors that could enhance its diagnostic precision, thereby increasing its overall accuracy and effectiveness in diverse populations.

## Conclusions

The SCORED questionnaire demonstrates low diagnostic accuracy for CKD, primarily due to its low specificity and PPV. However, its high sensitivity and NPV make it an effective initial screening tool for identifying individuals at high risk for CKD, particularly in asymptomatic populations. This is crucial in resource-limited settings where access to comprehensive diagnostic tests may be restricted.

Our study confirms that glycemic control is a significant factor in CKD risk stratification, highlighting the critical role of managing DM in preventing the onset and progression of CKD. This finding underscores the importance of integrated care approaches for patients with chronic conditions such as diabetes.

The ease of use, low cost, and high sensitivity of the SCORED questionnaire make it an appealing option for widespread screening initiatives. Our results corroborate those of earlier studies, including the validation study conducted by Magacho and colleagues in Brazil. By reinforcing the validity of the Brazilian version of the SCORED questionnaire, we contribute to the growing body of evidence supporting its utility in diverse demographic settings.

In conclusion, while the SCORED questionnaire should not be solely relied upon for definitive CKD diagnosis, it is a valuable tool for preliminary screening. Its application can lead to early detection and timely intervention, which are pivotal in mitigating the long-term impacts of CKD. Future studies should focus on improving its specificity and exploring additional factors that could enhance its diagnostic precision.
